# hsa-let-7c miRNA Regulates Synaptic and Neuronal Function in Human Neurons

**DOI:** 10.3389/fnsyn.2018.00019

**Published:** 2018-07-17

**Authors:** Heather McGowan, Vincent R. Mirabella, Aula Hamod, Aziz Karakhanyan, Nicole Mlynaryk, Jennifer C. Moore, Jay A. Tischfield, Ronald P. Hart, Zhiping P. Pang

**Affiliations:** ^1^Child Health Institute of New Jersey, New Brunswick, NJ, United States; ^2^Department of Neuroscience and Cell Biology, Rutgers University, Piscataway, NJ, United States; ^3^Department of Genetics, Rutgers University, Piscataway, NJ, United States; ^4^Department of Cell Biology and Neuroscience, Rutgers University, Piscataway, NJ, United States

**Keywords:** non-coding RNA, microRNA, synaptic transmission, human neurons, stem cells

## Abstract

Non-coding RNA, including microRNA (miRNA) serves critical regulatory functions in the developing brain. The let-7 family of miRNAs has been shown to regulate neuronal differentiation, neural subtype specification, and synapse formation in animal models. However, the regulatory role of human let-7c (hsa-let-7c) in human neuronal development has yet to be examined. Let-7c is encoded on chromosome 21 in humans and therefore may be overexpressed in human brains in Trisomy 21 (T21), a complex neurodevelopmental disorder. Here, we employ recent developments in stem cell biology to show that hsa-let-7c mediates important regulatory epigenetic functions that control the development and functional activity of human induced neuronal cells (iNs). We show that overexpression of hsa-let-7c in human iNs derived from induced pluripotent stem (iPS), as well as embryonic stem (ES), cells leads to morphological as well as functional deficits including impaired neuronal morphologic development, synapse formation and synaptic strength, as well as a marked reduction of neuronal excitability. Importantly, we have assessed these findings over three independent genetic backgrounds, showing that some of these effects are subject to influence by background genetic variability with the most robust and reproducible effect being a striking reduction in spontaneous neural firing. Collectively, these results suggest an important function for let-7 family miRNAs in regulation of human neuronal development and raise implications for understanding the complex molecular etiology of neurodevelopmental disorders, such as T21, where let-7c gene dosage is increased.

## Introduction

Synaptic transmission mediates fast information flow in the brain. As such, synapses represent the fundamental unitary elements of neuronal circuits and brain network systems that ultimately govern all behavior and cognitive processes. Clinical manifestations of defective synaptic transmission include, but are not limited to, psychiatric illness, such as schizophrenia, bipolar disorder and major depression; developmental disorders, such as autism; and cognitive difficulties, such as intellectual disability (van Spronsen and Hoogenraad, 2010). Synaptic transmission and neuronal function is highly regulated by various factors, including non-coding miRNAs, which constitute an important epigenetic regulatory system affecting gene expression at the post-transcriptional level (Olde Loohuis et al., 2012). Nevertheless, how miRNAs impact neuronal structure and function, especially at the level of synaptic transmission in human neurons, remains enigmatic.

MicroRNAs (miRNA) are suggested to play important roles in neurogenesis, cell migration, neuronal maturation, dendritic arborization, axonal regeneration, and, importantly, synaptic development and plasticity in the brain (Giraldez et al., 2005; Weston et al., 2006; Bak et al., 2008; Edbauer et al., 2010; Zou et al., 2013; Giusti et al., 2014). Among the most highly expressed miRNAs in the mammalian brain are members of the lethal-7 (let-7) family (Lagos-Quintana et al., 2002). Let-7 miRNAs were among the first discovered in *C. elegans* (Roush and Slack, 2008) and are highly conserved across species, with important roles in temporally-regulated developmental processes, such as terminal differentiation and cell specification, as well as cell cycle regulation and tumor suppression (Roush and Slack, 2008). In the developing brain, let-7 miRNAs are involved in regulating many cellular functions, including neuronal differentiation (Schwamborn et al., 2009), neural cell subtype specification (Wu et al., 2012), neuronal regeneration (Zou et al., 2013; Li et al., 2015), and synapse formation (Caygill and Johnston, 2008; Edbauer et al., 2010). Although computational modeling has implicated the let-7 gene family in the regulation of post-synaptic transcripts (Paschou et al., 2012), little is known about what, if any, direct functions let-7 family members regulate in mature, human neuronal cells. Moreover, hsa-let-7c is encoded by chromosome 21 (HSA21) and is present in an extra copy in Trisomy 21 (T21), a variable and complex neurodevelopmental syndrome characterized by symptoms including mild to moderate intellectual disability (Antonarakis, 2017). Indeed, recent reports have suggested that HSA21-encoded miRNAs may be important to fully understand T21-associated disease pathophysiology (Klusmann et al., 2010; Keck-Wherley et al., 2011; Izzo et al., 2017). Therefore, we sought to define the neuronal and synaptic regulation exerted by let-7c, among the most highly conserved HSA21-encoded miRNAs, in a human neuronal context.

Using human iN cells (Zhang et al., 2013) derived from induced pluripotent stem (iPS) cells and embryonic stem (ES) cells as a model system, we investigated the impact of hsa-let-7c on neuronal morphologic maturation, spontaneous and evoked action potentials (APs), as well as synapse formation and synaptic strength. We found that lentiviral-mediated over expression of hsa-let-7c impairs neuronal and synaptic development and markedly reduces neuronal excitability. Furthermore, we assessed these functions over three different pluripotent cell lines derived from independent genetic backgrounds, revealing a consistent role for let-7c in regulating spontaneous neuronal firing activity. These analyses also show that some features of epigenetic regulation by let-7c may be subject to regulation by genetic background or epigenetic state, as only two of the lines analyzed showed a statistically significant synaptic phenotype and intrinsic excitability deficit. Nevertheless, these results implicate let-7c as an important regulator in human neuronal development and function, and thus shed light onto possible epigenetic regulatory mechanisms associated with gene-dosage dependent pathophysiology mediated by non-coding RNAs associated with T21 in humans.

## Materials and Methods

### Stem Cell Culture and Rapid Neuronal Induction

Two lines of control iPS cells were obtained for use in this study. One line (AG2U) was obtained from the Bhattacharyya Lab at the University of Wisconsin and was derived from the male fibroblast line AG05397 (Coriell; Weick et al., 2013). The other pair T21C1 (ND50026) and T21C5 (ND50027) is a female line that was obtained from the NINDS Human Cell and Data Repository housed at RUCDR Infinite Biologics^®^. T21C1 iPS cell clones were reprogrammed from the female fibroblast line AG06872 (Coriell) using retrovirus. The T21 karyotype reverted to normal during culture, producing a euploid isogenic clone (T21C5, referred as CRM27 in this study), which we have utilized in this study. In addition, we utilized the well-characterized H1 human ES cell line (NIH registry 0043) for this study, which has been described previously (Thomson et al., 1998). The use of ES cells was approved by the Rutgers University ES Cell Oversight (ESCRO) Committee. Mouse glial cells were used in this study. All protocols involving using animals were approved by Institutional Animal Care and Use Committee (IACUC) at Rutgers.

iPS and ES cells were cultured feeder cell-free (Xu et al., 2001) on Corning™ Matrigel™ Membrane Matrix (Thermo Fisher)-coated 35 mm dishes in mTeSR™1 medium (Stem Cell Technologies). The medium was refreshed daily, and the cultures were passaged as single cells every 4–6 days. Briefly, prior to passage, 35 mm dishes (or, alternatively, individual wells of a 6-well plate) were coated with 1 mL of Matrigel™ and incubated at 37°C for 30–60 min. Residual Matrigel™ was removed just prior to addition of the dissociated cells. The culture medium was aspirated, and the plates were washed with 1 mL Minimum Essential Medium (MEM; Gibco). The MEM was then replaced with 1 mL Accutase™ (Stem Cell Technologies) and incubated for 3 min at 37°C to dissociate the cells. The cells were collected with the Accutase™ into a 15 mL conical tube. Plates were washed with 1 mL MEM to collect residual cells, which was then added to the conical tube. The cells were pelleted in a tabletop centrifuge at 1000 RPM for 5 min, and the supernatant was removed. The cells were resuspended in 1 mL mTeSR™1 and 5 μM ROCK inhibitor (Stemolecule™ Y27632, Stemgent). Then 10 μl of the cell solution was diluted 1:10, and 10 μl of this diluted cell suspension was loaded into a hemocytometer for counting. Once the concentration of cells was determined, the original cell suspension was diluted such that each new 35 mm maintenance dish would contain 250,000 cells. The diluted cell suspension was added drop-wise to a Matrigel™ coated 35 mm dish or 6-well plate containing 1.5–2 mL mTeSR™1 and 5 μM ROCK inhibitor.

To generate human iN cells, iPS or ES cells were infected with a lentiviral cocktail consisting of mTeSR™1, 5 μM ROCK inhibitor, viruses packaged with the transcription factor Neurogenin2 (Ngn2) and the reverse tetracycline-controlled transactivator (rtTA), as described previously (Zhang et al., 2013). The following day, the lentiviral cocktail was removed and replaced with Neurobasal^®^ (Gibco) medium containing Gem21 NeuroPlex™ Serum Free Supplement (Gemini), L-glutamine (Gibco), 2 μg/ml doxycycline (MP Biomedicals), and 5 μM ROCK inhibitor. Selection with 1 μg/ml puromycin was performed as needed following 24 h of induction with doxycycline. On day 5 of induction, cells were dissociated with Accutase^®^ and replated using neuronal culture medium containing Neurobasal^®^, L-glutamine, Gem21, 2 μg/ml doxycycline, 10 ng/ml BDNF, 10 ng/ml GDNF, 10 ng/ml NT3, and 5% fetal bovine serum (FBS; Atlanta Biologicals) and seeded onto glass coverslips with a monolayer of mouse glial cells. After replating, 50% neural culture medium was exchanged every 3–4 days, containing 2 μM cytosine arabinoside to inhibit glial overgrowth. Morphological and electrophysiological analyses were conducted 4–5 weeks following initial induction with doxycycline.

### Cloning of MECP2 3’UTR Luciferase Reporter System

For luciferase experiments, we cloned a portion of the 3’UTR of MECP2 from human genomic DNA isolated from iPS cells using PCR. The fragment of the MECP2 3’UTR containing the miRNA targeting sites of interest was amplified via PCR using the following primers: (sense: 5’-ctcgagTTGTGAACAGCAGAATTGACCGAC-3’); (antisense: 5’-gcggccgcGGAGCAAACACAGCACACGTTAC-3’) and inserted into the PsiCHECK2 vector (Promega™), downstream of the Renilla luciferase reporter using NotI and XhoI sites and confirmed by Sanger sequencing.

### Cloning and Validation of Lentiviral Let-7c Overexpression Constructs

To create the hsa-let-7c short hairpin overexpression construct, we designed oligonucleotides containing the mature let-7c sequence and its reverse sequence, separated by an artificial loop sequence, and flanked by BglII and HindIII restriction enzyme sequences: (sense, 5’-gat cccgTGAGGTAGTAGGTTGTATGGTTCTCAAGAGAAACCA TACAACCTACTACCTCATTTTTa-3’); (antisense, 5’-agcttAAAAATGAGGTAGTAGGTTGTATGGTTTCTCTTGAGAACCAT ACAACCTACTACCTCAcgg-3’), which were cloned downstream of the H1 promoter in the pSUPER RNAi vector (Oligoengine). The resulting pSUPER-let-7c construct then underwent a second round of cloning in order to transfer the H1 promoter and let-7c short hairpin from pSUPER into the lentiviral backbone FG12.

HEK293T cells were transfected with either an empty FG12 backbone (Control) or the FG12-let-7c overexpression construct (Let-7c) via calcium phosphate transfection. Cells were harvested 48 h later and RNA was isolated using the *mir*Vana miRNA Isolation kit (Ambion™) according to the manufacturer’s instructions to yield a pool of RNA enriched for small RNAs (referred to here as “miRNA”). For qRT-PCR, 10 ng of isolated miRNA was used for reverse transcription (RT) reaction using the TaqMan™ MicroRNA RT Kit (Applied Biosystems™) on the GeneAmp^®^ PCR System 9700 (Applied Biosystems™) thermal cycler. Expression levels were determined via qPCR using TaqMan™ MicroRNA Assays (Applied Biosystems™) on a QuantStudio™ 6 Flex Real-Time PCR System (Applied Biosystems™). We used the ΔΔC_T_ (cycle threshold)-method for relative gene expression quantification as described previously (Livak and Schmittgen, 2001).

### Immunocytochemistry and Imaging Analysis

Cells were fixed in 4% paraformaldehyde for 10 min at room temperature. After fixation, cells were incubated in 0.1% Triton X-100 in PBS for 5 min at room temperature, then washed three times in phosphate buffered saline (PBS) and blocked in 4% bovine serum albumin and 1% goat serum for 30 min at room temperature. Primary antibodies were applied for 1 h at room temperature, followed by three washes with PBS. Secondary antibodies were applied for 30 min at room temperature, washed three times in PBS, and a final wash in deionized water. Coverslips were then mounted onto glass slides with Mounting Media containing DAPI (Fluoroshield™). The following antibodies were used for our analysis: mouse anti-MAP2 (Sigma, 1:1000), rabbit anti-MAP2 (Sigma, 1:1000), rabbit anti-synapsin (E028, 1:3,000), mouse anti-vGlut1 (Synaptic Systems, 1:200), mouse anti-vGAT (Synaptic Systems, 1:200), rabbit anti-TUJ1 (Synaptic Systems, 1:200), anti-TRA-1-60 (Millipore, 1:1000), anti-Oct4 (Millipore, 1:1000) and anti-MeCP2 (Neuromab, 1:500). Secondary antibodies were diluted at 1:500 (Alexa 488, Alexa 546, Alexa 633, Molecular Probes). Images were taken on a Zeiss LSM700 confocal microscope.

For synapsin quantification, 63× images were taken of cells that were positive for both GFP (indicative of infection with FG12 or FG12-let-7c lentiviral constructs) and MAP2. For iPS cell-derived iN cells, a secondary dendrite was cropped from each image using Photoshop. The number of synapsin^+^ puncta, as well as puncta size and fluorescence intensity of synapsin staining, were blindly identified using our recently developed *Intellicount* high-throughput synapse quantification program (Fantuzzo et al., 2017). All imaging and staining procedures were performed blind to experimental condition.

### Functional Analysis by Electrophysiology

Whole-cell patch clamp experiments were performed as described elsewhere (Vierbuchen et al., 2010; Pang et al., 2011b). After 28 days of post-induction with doxycycline, coverslips were transferred to a recording chamber for performing electrophysiology using a Multiclamp 700B amplifier (Molecular Devices, CA, USA). The bath solution contained (in mM): 140 NaCl, 5 KCl, 2 MgCl_2_, 2 CaCl_2_, 10 HEPES, 10 glucose (pH 7.4, adjusted with NaOH). The whole-cell pipette solution contained (in mM): 126 K-Gluconate, 4 KCl, 10 HEPES, 0.3 Na-GTP, 4 Mg-ATP and 10 Phosphocreatine (pH 7.2, adjusted with KOH). After successfully gaining access for whole cell recordings, passive membrane properties, including the membrane input resistance (Rm) and membrane capacitance (Cm), resting membrane potential (RMP) were recorded. Spontaneous excitatory post-synaptic currents (sEPSCs) were obtained under voltage clamp recording mode at a holding potential of −70 mV. Recordings of spontaneous AP firing were performed in current clamp mode with no external current injected, while evoked APs were recorded by holding the membrane potential around −65 mV and injecting current ramps (0–200 pA, over a 500 ms duration) or 5 pA stepwise currents to measure neuronal excitability. Data were collected using Clampex 10.5 (Molecular devices) acquisition software. Current responses were recorded using a low-pass filter at 2 kHz and a sampling rate of 5 (Figures 2, 3) or 10 kHz (Figure 4). Clampfit 10.5 (Molecular Devices) was used for offline analysis of the data.

**Figure 1:**
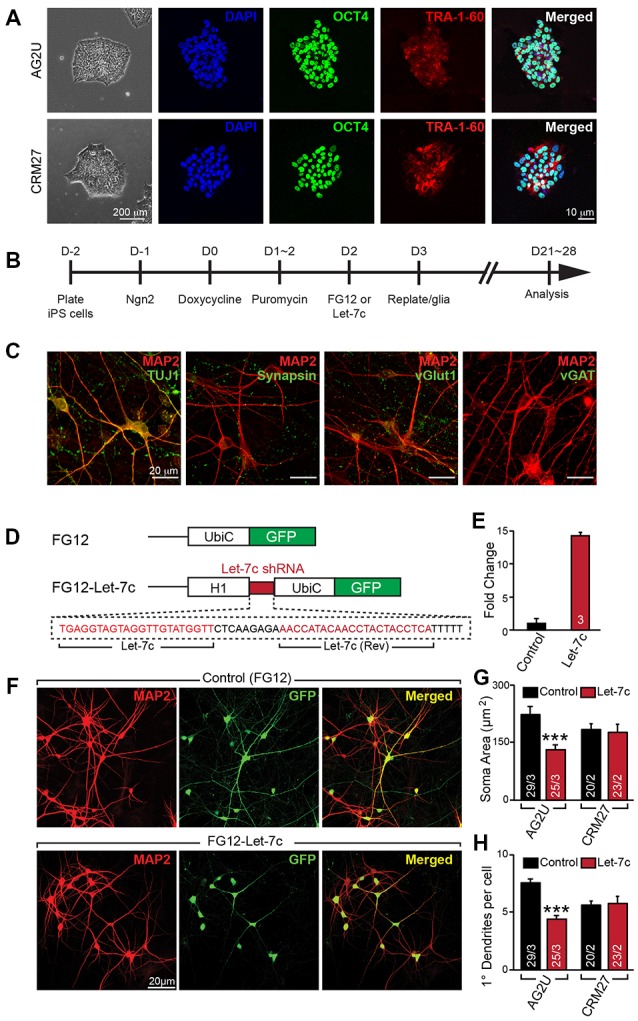
Hsa-let-7c overexpression alters human induced neuronal (iN) cell morphology. **(A)** induced pluripotent stem (iPS) cells stain positive for pluripotent markers OCT4 and Tra-1-60. **(B)** iN cell induction protocol for converting human iPS cells into functional glutamatergic neurons. **(C)** Immunocytochemical characterization of excitatory iN cells. iN cells transduced with Ngn2 stain positive for the neuronal lineage marker TUJ1, mature neuronal marker MAP2, and presynaptic marker synapsin; Ngn2-iN cells are also positive for the excitatory synaptic marker, vGlut1, and negative for the inhibitory marker vGAT. **(D)** Design for the let-7c overexpression construct. The H1 promoter and a downstream short hairpin RNA (shRNA) containing the mature let-7c sequence were cloned into the FG12 lentiviral backbone. **(E)** HEK293T cells were transfected with either FG12 or FG12-let-7c and assayed via qPCR to assess let-7c overexpression. **(F–H)** The FG12-let-7c construct was packaged into lentivirus and used to infect human iN cells according to **(B)**. Infected cells express GFP **(F)** and AG2U cells show a significant reduction in soma size **(G)**, as well as primary dendrite outgrowth **(H)**. Statistical tests were performed using two-tailed Student’s *t*-test, where *p < 0.05, **p < 0.01 and ***p < 0.001.

**Figure 2:**
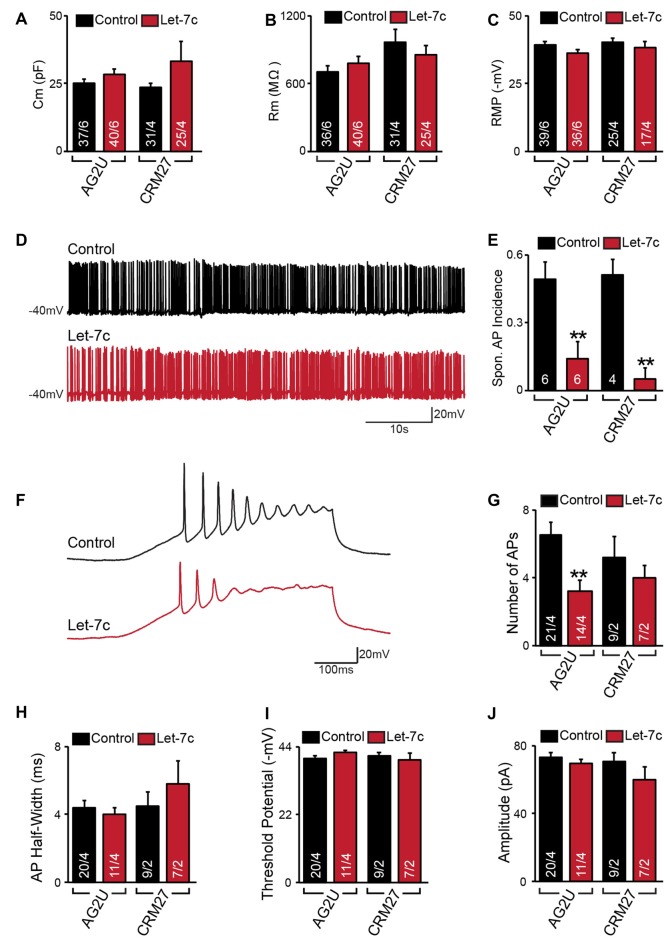
Hsa-let-7c overexpression reduces action potential (AP) firing. **(A–C)** let-7c overexpression did not alter membrane capacitance **(A)**, membrane resistance **(B)**, or Resting membrane potential (RMP) **(C)** of iN cells. **(D,E)** While iN cells overexpressing let-7c were capable of firing spontaneous APs **(D)**, a significantly smaller proportion of cells fired spontaneous APs (Spon. AP) compared to controls **(E)**. **(F,G)** iN cells overexpressing let-7c fired fewer APs per ramp compared to controls. **(H–J)** let-7c overexpression did not change the half-width **(H)**, threshold potential **(I)**, or peak amplitude **(J)** of APs induced by current ramp, suggesting that the reduction in AP firing is likely due to a reduction in excitability rather than maturity. Statistical tests were performed using two-tailed Student’s *t*-test, where **p < 0.01.

**Figure 3:**
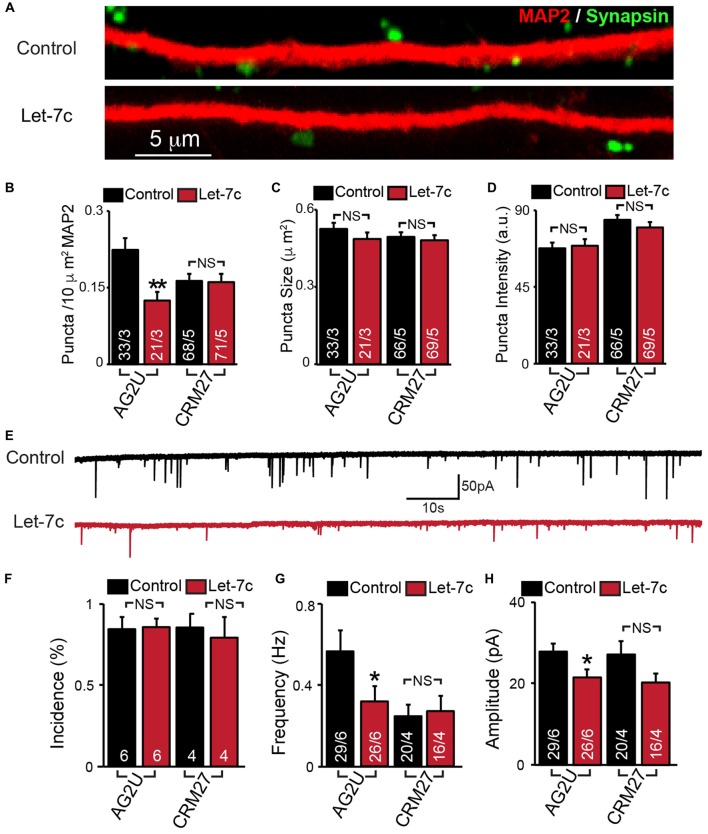
Hsa-let-7c overexpression alters synaptic transmission in human iN cells. **(A–D)** let-7c overexpression significantly reduced the density but not size or fluorescence intensity of synapsin^+^ puncta in AG2U iPS-derived induced neurons. **(E)** Representative traces of spontaneous excitatory post-synaptic currents (sEPSCs) in control and let-7c infected iN cells. **(F)** Overexpression of let-7c does not decrease the incidence of sEPSCs in either cell line. **(G)** let-7c overexpression results in a significant decrease in sEPSC frequency in AG2U iPS-derived induced neurons. **(H)** The amplitude of sEPScs is significantly decreased by let-7c overexpression in the AG2U cell line. Statistical tests were performed using two-tailed Student’s *t*-test, where *p < 0.05, **p < 0.01.

**Figure 4:**
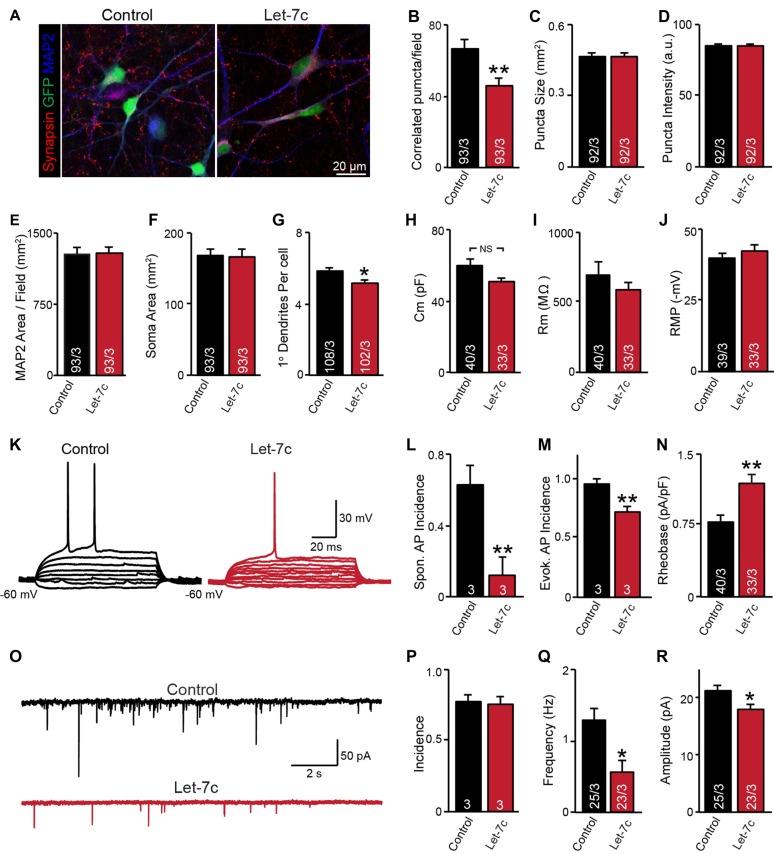
let-7c overexpression impairs neuronal development and synaptic function in H1 embryonic stem (ES) cell-derived human induced neurons. **(A)** Representative image of neurons expressing control or hsa-Let7c lentiviruses (green). **(B–D)** MAP2-correlated synapse density and puncta characteristics display a selective decrease in number of synaptic puncta. **(E–G)** Neuronal soma size, primary dendrite formation and total segmented MAP2-positive area reveal a mild impairment of primary dendrite outgrowth. **(H–J)** Intrinsic membrane properties measured under voltage-clamp at −70 mV (capacitance and input resistance) or current clamp (RMP) are not altered. **(K)** Representative traces of stepwise current injection protocol for evoked AP measurement of neuronal excitability. **(L–N)** Spontaneous and evoked AP incidence and rheobase values display a marked reduction in cell excitability. **(O)** Representative traces of spontaneous postsynaptic currents (sPSCs) measured at −70 mV. **(P–R)** Summary data for incidence (proportion of cells with detectable responses) frequency, and amplitude of sPSCs. All data are displayed as mean ± SEM and represent the results of three independent experiments. Statistical tests were performed using two-tailed Student’s *t*-test, where *p < 0.05 and **p < 0.01.

### Statistical Analysis

All data are presented as the mean ± standard error of the mean (SEM), unless otherwise indicated. All experiments were repeated on at least three biological replicates, with each replicate consisting of an independent differentiation and/or culture passage. Data were excluded as outliers if their modified Z score, as determined by Iglewicz and Hoaglin’s robust test for multiple outliers, was greater than 3.5. Outlier detection was accomplished using an online calculator found at http://contchart.com/outliers.aspx. Statistical significance was determined by Student’s *t*-test or Mann-Whitney U test, as determined by the Shapiro-Wilk test. Goodness-of-fit analysis utilizing the Shapiro-Wilk test was performed using an online calculator found at http://contchart.com/goodness-of-fit.aspx.

## Results

### hsa-let-7c Overexpression Alters Morphological Development in Human iNs

To investigate the role of let-7c in human neuronal development and function, we derived excitatory glutamatergic iN cells from human iPS cells utilizing forced expression of the transcription factor Ngn2 (Zhang et al., 2013). We utilized euploid control iPS cell lines AG2U and CRM27, which exhibited polarization and neuronal morphology upon neuronal induction (Figure 1A). Expression of Ngn2 from the virally-delivered transgene was induced with doxycycline, and infected cells were selected using puromycin (Figure 1B). The resulting mature iN cells stained positive for the neuronal markers TUJ1 and MAP2, as well as the presynaptic markers synapsin and vGlut1, but negative for the inhibitory presynaptic marker, vGAT (Figure 1C).

We constructed an H1 promoter-driven short hairpin RNA (shRNA) to express the mature let-7c sequence (Figure 1D) in the FG12 vector backbone (Qin et al., 2003). In each of our experiments we utilized this FG12-let-7c (“Let-7c”) construct to drive let-7c overexpression, alongside an empty FG12 vector control, which is in line with many previous shRNA studies (Edbauer et al., 2010; Pang et al., 2010a,b, 2011a; Zhou et al., 2013). To verify the construct for let-7c expression, we transfected HEK 293T cells with either the Let-7c or the empty vector control construct and measured let-7c levels by qPCR, finding a ~14 fold increase in the cells transfected with the overexpression construct (Figure 1E).

We next infected both AG2U and CRM27 iN cells with viruses before re-plating them onto a monolayer of glial cells (Figure 1B). The FG12 backbone also contains a Ubiquitin C promoter-driven GFP reporter, which allowed us to identify iN cells infected with Control or Let-7c viruses (Figures 1D,F).

Interestingly, the AG2U-iNs overexpressing let-7c exhibit both a reduction in soma size (Figures 1F,G) and primary dendrite formation (Figures 1F,H and Supplementary Table S1), however, this was not the case in CRM27-iN cells. Note that AG2U is a male iPS line while the CRM27 line was derived from a female subject. We speculate that different genetic backgrounds or sex of origin might impact the outcomes of let-7c overexpression on human neuronal development. Nevertheless, these data highlight the importance of examining phenotypes over multiple genetic backgrounds and sexes.

### let-7c Overexpression Reduces Spontaneous Neuronal Firing and Intrinsic Excitability

We next sought to determine what effects, if any, let-7c overexpression has on functional activity of human iN cells. At 4–5 weeks post induction with doxycycline, we assessed the effect of let-7c overexpression on neuronal function using whole cell patch clamp recording, selecting only neurons showing clear green fluorescence for analysis. We found that, compared to control cells, AG2U- and CRM27-iN cells that overexpress let-7c do not exhibit any significant changes in intrinsic electrical membrane properties, including membrane capacitance, input resistance, or RMP (Figures 2A–C). However, AG2U- and CRM27-iN cells that overexpress let-7c were significantly less likely to fire spontaneous APs (Figures 2D,E). Moreover, when iN cells were induced to fire APs using a current ramp protocol, we found that let-7c overexpression in the AG2U-iN cells led to a reduction in the number of APs fired during current injection (Figures 2F,G), an effect which was not significant in the CRM27 iN cells. We also investigated the kinetic and threshold properties of evoked APs and found no change in the half-width (Figure 2H) or firing threshold in either cell line (Figure 2I). Likewise, we found no difference in the amplitude of evoked APs (Figure 2J, Supplementary Table S2). To further examine the possibility of intrinsic excitability differences, we recorded voltage-dependent whole-cell sodium and potassium currents, finding a selective impairment in sodium conductance in the AG2U, but not in CRM27 cell line (Supplementary Figure S1). Collectively, these results suggest that hsa-let-7c may regulate spontaneous neural firing independent of other changes in the development of passive electrical membrane properties.

### hsa-let-7c Overexpression Alters Synaptic Transmission

To determine whether let-7c regulates synaptic connectivity, we measured the number of synapses in iN cell cultures overexpressing let-7c (Figure 3A). We found that let-7c overexpression led to a decrease in the density (Figure 3B), but not size or average fluorescence intensity (Figures 3C,D), of synapsin-positive puncta in AG2U-iN cells, without change in any of these parameters in the CRM27 line.

To examine the potential synaptic consequences of let-7c overexpression, we assessed the functional connectivity of these iN cells using whole cell patch clamp and recorded sEPSCs (Figure 3E). We first determined the percentage of cells showing detectable synaptic responses, and found no differences (Figure 3F). This suggests that let-7c overexpression does not grossly impair the development of functional synaptic connections. For AG2U-iN cells, there was a statistically significant reduction in the frequency of sEPSCs (Figure 3G), which was consistent with the reduction in density of synapses. AG2U-iN cells also exhibited a modest decrease in the amplitude of sEPSCs. Neither of these parameters, however, was significantly changed in CRM27-iN cells, in agreement with the lack of effect on synapse formation for this cell line (Figures 3B–D, Supplementary Table S3). Thus, let-7c regulates synaptic strength and connectivity in human neurons, a function that can be influenced by genetic or other factors.

### hsa-let-7c Impairs Neuronal Excitability and Synaptic Function in Human ES Cells

Although it is clear that overexpression of let-7c suppresses spontaneous spike firing, there were clear differences in synapse formation and intrinsic excitability between cell lines. To test whether the phenotypes observed in the AG2U cell line are unique, we additionally employed a well characterized and widely used human ES cell line (H1, male) for further analyses. Consistent with the AG2U cell line, we observed a significant decrease in the number of MAP2-correlated synapsin-positive puncta (Figures 4A,B) and no difference in the mean area or averaged fluorescence intensity of puncta between conditions (Figures 4C,D). As culture density has the potential to skew synapse formation results, we further quantified the MAP2 area present within each corresponding field, finding no differences between conditions (Figure 4E). Finally, we assessed soma size and primary dendrite outgrowth finding a mild but significant reduction in primary dendrite number (Figures 4F,G).

We next examined neuronal function using whole cell patch clamp. Consistent with our previous results with AG2U and CRM27 cells lines, there was no significant difference between Let-7c and Control H1-iN cells in intrinsic membrane properties measured under voltage clamp at −70 mV (Figures 4H–J). However, we did observe that H1-iN cells exhibit a dramatic reduction in excitability, as evaluated by the incidence of spontaneous AP firing, as well as APs that were evoked using small 5 pA steps of current injection (Figures 4K–N). For evoked AP firing, we calculated the percentage of cells firing an AP with 100 pA or less current injection and additionally measured the minimal current required to trigger the first AP (i.e., rheobase value), which was increased by let-7c overexpression (Figures 4M,N). This suggests that the reduction in excitability that we previously observed is present in at least one other cell line and may be due a specific impairment of AP firing in response to depolarizing current stimulation, a mechanism consistent with our observation of impaired voltage-dependent sodium conductance (Supplementary Figure S1).

Finally, we assessed functional connectivity using whole cell patch clamp to record sEPSCs (Figure 4O). Consistent with both iPS cell lines, there was no difference in the likelihood that H1-iN cells overexpressing let-7c received functional synaptic input compared to control cells (Figure 4P). Consistent with our AG2U functional data, as well as the reduction in the density of synapsin-positive puncta seen in both AG2U and H1-iN cells, there was a significant reduction in the frequency of sEPSCs with let-7c overexpression (Figure 4Q), as well as a mild decrease in the amplitude of sEPSCs (Figure 4R).

### let-7c Targets Epigenetic Regulator Methyl-CpG-Binding Protein 2 (MECP2)

In order to gain some insight into possible mechanisms by which overexpression of let-7c impairs neuronal development and synaptic function, we sought to identify a putative mRNA target of let-7c in human neurons. MECP2 is one possible candidate because it harbors a predicted let-7c targeting site within its 3’ UTR. Moreover, MECP2 is mutated in Rett syndrome (RTT), a neurodevelopmental disorder characterized by intellectual disability and some features related to autism spectrum-disorders (Amir et al., 1999) and both RTT mouse and human neuronal models show that, MECP2 deficient neurons have deficits in dendritic complexity and synaptic connectivity, as well as altered neuronal excitability (Jentarra et al., 2010; Marchetto et al., 2010; Xu et al., 2014).

**Figure 5:**
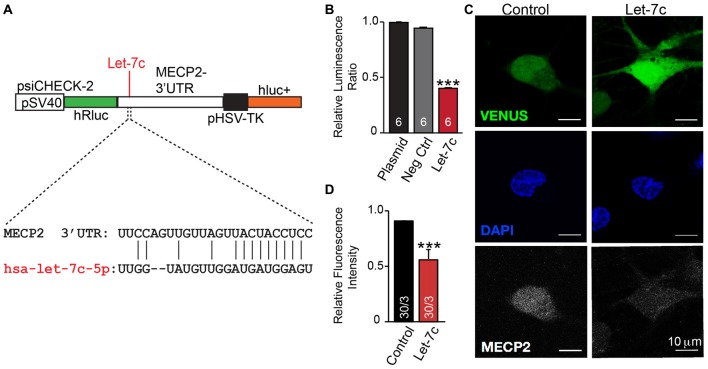
let-7c targets MECP2 3’ UTR and reduces MeCP2 protein levels in human induced neurons. **(A)** Schematic of the *MECP2* 3’UTR cloned into the psiCHECK-2 dual luciferase reporter, with indication of the putative hsa-let-7c binding site present within this sequence. **(B)** let-7c, but not control overexpression miRNA or empty vector, significantly reduces normalized Renilla luminescence compared to controls, suggesting that it is capable of binding to the *MECP2* 3’UTR. **(C,D)** Overexpression of hsa-let-7c in human iN cells results in a reduction in the relative fluorescence intensity of MeCP2 as determined by immunohistochemistry. ***p < 0.001.

We thus hypothesize that hsa-let-7c regulates MECP2 gene expression. We designed a dual-luciferase reporter assay by placing the MECP2 3’ UTR sequence, containing the putative let-7c targeting site, downstream of a Renilla luciferase open reading frame (Figure 5A). This vector also contains a firefly luciferase reporter gene under control of the HSV promoter to allow normalization of data to transfection efficiency. We co-transfected this reporter plasmid into HEK 293T cells along with miRNA mimetics for hsa-let-7c and observed a significant decrease in normalized Renilla luminescence, indicating that let-7c is capable of targeting this isolated fragment of *MECP2* 3’UTR to down-regulate protein translation (Figure 5B). Importantly, co-transfection with a commercially-available miRNA mimetic negative control did not change relative Renilla luminescence, showing specificity of let-7c (Figure 5B). In order to determine whether let-7c can regulate MeCP2 levels in human neurons, we infected iN cells with our Let-7c or control constructs, stained for MeCP2 by IHC and measured the fluorescence intensity in the nucleus. We found that iN cells overexpressing let-7c demonstrate a significant reduction in fluorescence intensity compared to controls (Figures 5C,D), suggesting that overexpressed let-7c is capable of down regulating MeCP2 expression in human iN cells.

## Discussion

In this study, we investigated the role of hsa-let-7c in human neuronal and synaptic development by overexpressing the mature let-7c sequence in pluripotent stem cell-derived induced neuronal cells. We have discovered that let-7c overexpression leads to a number of deficits in neuronal morphology, electrical excitability, and synaptic transmission, the most prominent being a striking reduction in spontaneous neuronal firing. Overall, our findings suggest that let-7c miRNA regulates human neuronal differentiation and function, in line with the established role for the let-7 miRNA family in developmental timing, regulation of the cell cycle, and promotion of differentiation (Roush and Slack, 2008). Here, we will discuss how let-7c may regulate neuronal development, and provide possible implications to consider for the etiology of neurodevelopmental disorders.

### hsa-let-7c and Neurite Outgrowth

Regulation of the actin cytoskeleton is essential to dendritic arborization, and the let-7 family of miRNA appear to regulate many cytoskeletal changes that influence cell motility and morphology (Huang et al., 2012; Hu et al., 2013; Zou et al., 2013). In this study, we observed that overexpression of hsa-let-7c in human iN cells can decrease primary dendrite outgrowth. There are a number of potential mechanisms supported by the literature that may explain a reduction in dendrite formation in response to let-7c overexpression. For example, the let-7 miRNAs have been shown to directly target members of the actin cytoskeleton pathway, *PAK1*, *DIAPH2*, *RDX* and *ITGB8* (Hu et al., 2013), which in turn inhibit cell migration. Additionally, let-7c mediates a cell-autonomous, age-related decline in axon regenerative capability in *C. elegans* by directly targeting LIN-41, a factor that promotes axon regeneration (Zou et al., 2013). Moreover, BDNF signaling suppresses the processing of mature let-7 by promoting the expression of Lin-28, which in turn promotes dendritic arborization (Huang et al., 2012).

The expansion of the dendritic arbor is crucial to formation of the appropriate number of synapses and thus information flow in widely distributed neuronal circuits throughout the brain (Tronel et al., 2010). Reduced dendritic complexity is a hallmark of a number of neurodevelopmental disorders characterized by intellectual disability, including T21 and RTT (Takashima et al., 1989; Chapleau et al., 2009). Hsa-let-7c is encoded on HSA21, which is present in three copies in T21. As such, it may be overexpressed, which could lead to mis-regulated expression of target genes, or override signaling cascades that regulate its expression, as discussed above, which may in turn contribute to the reduced dendritic outgrowth.

Finally, we have shown that let-7c is capable of targeting the MECP2 3’ UTR and that the MeCP2 levels are reduced in iN cells overexpressing let-7c. RTT animal models as well as human neuronal models that have decreased or deficient MeCP2 results in impaired dendrite outgrowth as well as reduced synaptic connectivity (Jentarra et al., 2010; Marchetto et al., 2010; Kron et al., 2012; Xu et al., 2014). While further work will be needed to establish a causal relationship between let-7c and MECP2 in regulating human neuronal development, these data now show that let-7c can target the MECP2 3’ UTR and regulate its protein levels in human neuronal cells.

### hsa-let-7c and Neuronal Excitability

In this study, we observed a decrease in the likelihood of iN cells from all three cell lines to fire spontaneous APs. The intrinsic membrane properties and characteristics of the AP waveform (threshold potential, kinetics and amplitude) were unchanged in iN cells overexpressing let-7c. However, in addition to the reduced spontaneous firing, let-7c iN cells derived from the AG2U and H1 cell lines showed an overall reduction in excitability as measured by a reduction in the number of APs evoked using a whole cell patch clamp current ramp and an increase in the current required to elicit the first AP.

The reduction in spontaneous AP firing may reflect a combination of reduced intrinsic excitability, as well as the observed decrease in synaptic strength. It has been reported that artificially increasing the excitability of individual cells via expression of a bacterial voltage-gated sodium channel led to increases in their dendritic and axonal synaptic connections (Sim et al., 2013). Moreover, it has also been shown that MeCP2 deficient neurons in the forebrains of RTT mice model exhibited decreased levels of Fos expression, likely reflecting decreased baseline activity, relative to controls at 6 weeks of age, an effect that was not present at 3 weeks (Kron et al., 2012). We demonstrated that let-7c can target MECP2 (Figure 5), which in turn may alter intrinsic neuronal excitability as well as synapse formation (Jentarra et al., 2010; Marchetto et al., 2010; Xu et al., 2014). Interestingly, MeCP2 has been shown to be down regulated in T21 human neurons (Weick et al., 2013), a disorder that is also characterized by reductions in cellular excitability (Cramer et al., 2015). Given that let-7c is encoded by HSA21, it is plausible that increased expression of this miRNA could lower MeCP2 protein levels.

### hsa-let-7c Reduces Synaptic Strength

We observed a significant decrease in synaptic density, as well as a decrease in the overall frequency of sEPSCs in two of the three cell lines tested, indicating a relative decrease in functional synaptic connectivity. The observed reduction in synaptic connectivity is consistent with studies done in *Drosophila*, which have implicated let-7 in the maturation and growth of the neuromuscular junction (NMJ; Caygill and Johnston, 2008; Sokol et al., 2008), which is often used as a model for synapse development. Together with miR-125, let-7 promotes the architecture of the NMJ via regulation of the gene *abrupt*, which is known for its roles in NMJ targeting and dendritic arborization (Caygill and Johnston, 2008). A reduction in the number of synapses could also be related to the morphological data because reduced dendritic complexity could present fewer contact sites for presynaptic boutons. Thus, despite the relative paucity of studies demonstrating that let-7 has a direct role in synapse formation, our study has provided evidence that let-7c can negatively regulate the density of synapses.

In addition to the morphological changes we observed, overexpression of let-7c may result in a decrease in synaptic connectivity and strength. Human neurons derived from the AG2U iPS and H1 ES cell lines also demonstrated a significant reduction in the amplitude of spontaneous EPSCs, which is often due to a reduction in postsynaptic receptor content. While we did not test this directly, this putative mechanism is consistent with computational analyses predicting that let-7 targets map to the postsynaptic compartment (Paschou et al., 2012). The potential connection between excitability, morphology, and physiology alluded to by our data is further supported by other predicted let-7 targets that are known to regulate activity-dependent plasticity, a process that functions at the intersection of these three properties. For example, in response to neuronal activity and membrane depolarization, the influx of calcium into a neuron induces dephosphorylation of myocyte enhancer factor 2 (MEF2) by the predicted let-7 target calcineurin. This ultimately leads to an increase in expression of proteins that function within the synapse, such as Arc. Activity-dependent activation of Arc has been shown to decrease the number of excitatory synapses. In addition to potentially being directly targeted by let-7, calcineurin is also inhibited by DYRK1A (via calcipressin), a protein encoded by HSA21 in humans and overexpressed in T21 (Ebert and Greenberg, 2013). In this way, there may be cross talk between let-7 miRNA and the other regulatory mechanisms disrupted in T21.

### Caveats and Additional Considerations

Our results show that let-7c can decrease spontaneous AP firing across three independent cell lines and suggest an important role for let-7c in human neuronal development. However, some phenotypes were apparent in only two of the three cell lines tested. These results highlight the importance of examining multiple cell lines and that conclusions drawn from examining one genetic background may be misleading. Indeed, there can often be considerable baseline and phenotypic variation between cell lines derived from two individuals due to differences in genetic background as well as separate clones generated from a single individual (Liang and Zhang, 2013). Moreover, a recent report suggests that genetic mutations can accumulate with passages of iPS cells (Merkle et al., 2017). Supporting the idea that there are “line-to-line” differences, the control measurements in the CRM27 cell line were often lower compared to those in the AG2U and H1 ES cell line (e.g., EPSC frequency, synapse density and primary dendrite number). Thus the discrepancy in some of our results highlights the importance of repeating experiments across multiple cell lines. Our results also show clearly that this approach is needed to identify which aspects of a phenotype are least subject to background genetic variability or other factors. In our case, a marked decrease (>80%) in the propensity to fire spontaneous APs was clearly observed in all three cell lines. Thus, we were able conclude that development of spontaneous neuronal activity is strongly influenced by let-7c in human neurons, while synapse formation and neuronal morphology may be more variable and depend on other factors.

Furthermore, while the use of overexpression experiments has been widely employed for ascribing cellular functions to individual miRNA (Edbauer et al., 2010; Mishima et al., 2016), the possibility of overexpression artifacts is always present. For example, forced stoichiometry between the miRNA and potential binding sites that may not have biological relevance under physiological conditions (Thomson et al., 2011) or an overabundance of pre-miRNA (or in our case, shRNA) that could potentially overload the miRNA processing machinery and displace other miRNA from Dicer or RISC complexes (Khan et al., 2009). We utilized a lentivirus prepared from an empty vector backbone as a sham to control for any changes that could occur with lentiviral infection and transgene integration. The weakness in this design is that it does not control for the potential overloading of the miRNA processing machinery. However, we don’t conclude that this invalidates the entire study as many functional and morphometric parameters were consistently unchanged between conditions. For example, despite the decreased neuronal excitability, the properties of the APs (e.g., amplitude, half-width, etc.) as well as the intrinsic membrane properties of iN cells remained unchanged. This would imply that a particular regulatory mechanism that controls development of spontaneous spike firing is specifically impaired by increased let-7c levels. Moreover, let-7c overexpression reduced the number of synaptic connections in two cell lines, but did not alter the size or intensity of synaptic puncta in any of the three cell lines tested, again implying a specific rather than a general impairment associated with our findings. Finally, control of overexpression of miRNAs could be difficult, and many previous studies involving shRNAs utilized vector as control (Edbauer et al., 2010; Pang et al., 2010a,b, 2011a; Zhou et al., 2013).

In summary, hsa-let-7c is a highly conserved, non-coding RNA that is abundantly expressed in the developing nervous system, with important roles in proliferation and differentiation. Here, we have shown that let-7c may play a pivotal role in human neuronal development and synaptic function in human neuronal cells. These findings, together with the chromosomal location of let-7c on HSA21 suggests its potential pathogenic role in the development of T21, warranting further investigation.

## Author Contributions

HM conducted iPS cell experiments, miRNA measurements and MeCP2 dual-luciferase assays. VM conducted H1 ES cell experiments and assisted with iPS neuronal differentiations together with AH. AH conducted immunocytochemistry and confocal imaging and provided technical support. AK performed molecular cloning of the *MECP2* 3’UTR and NM conducted MeCP2 immunocytochemistry and imaging. HM and VM analyzed data and wrote the manuscript with ZP. JM, JT, RH provided research materials and contributed to overall data interpretation and manuscript editing. ZP conceived overall aim of the study.

## Conflict of Interest Statement

The authors declare that the research was conducted in the absence of any commercial or financial relationships that could be construed as a potential conflict of interest.
