# Supplementary material for: hsa-let-7c miRNA Regulates Synaptic and Neuronal Function in Human Neurons
Source: Front Synaptic Neurosci. 2018 Jul 17;10:19. doi: 10.3389/fnsyn.2018.00019 (PMC6056636; doi:10.3389/fnsyn.2018.00019)
Supplement: Supplementary file 1 [file Presentation_1.PDF]

Supplementary Information

hsa-let-7c miRNA regulates synaptic and neuronal function in human neurons
McGowan et al.

Statistical data presented in the manuscript are as the following.

Table S1. Statistical values for Let-7c overexpression experiments using the AG2U cell line. AP = Action Potential, Syn = Synapsin, EPSC = excitatory post-synaptic current. N: numbers of neurons/ batches of cultures

	Control			Let-7c			
	Mean	SEM	N	M	SEM	N	p
Soma Size (μm^2)	224	20.7	29/3	131	12.3	25/3	3.8×10^{-4}
Number of 1° Dendrites	7.6	0.36	29/3	4.4	0.34	25/3	4.5×10^{-8}
Spontaneous AP Incidence	0.49	0.08	6	0.14	0.07	6	0.008
Number of Evoked APs	6.5	0.75	21/4	3.2	0.64	14/4	0.003
Evoked AP Half-width (ms)	4.4	0.43	21/4	4.0	0.39	11/4	0.51
Evoked AP Firing Threshold (-mV)	40	0.88	20/4	42	0.89	11/4	0.21
Evoked AP Amplitude (pA)	73.2	2.47	20/4	69.3	2.39	11/4	0.27
Syn^+ puncta per $10 \mu m^2$ dendrite	2.23	0.23	31/3	1.25	0.16	21/3	6.45×10^{-5}
Syn^+ Puncta Size (μm^2)	0.52	0.024	33/3	0.49	0.024	21/3	0.30
Syn^+ Puncta Intensity (a.u.)	67.7	1.25	33/3	69.2	1.84	21/3	0.47
Spontaneous EPSC Incidence	0.85	0.07	6	0.87	0.05	6	0.85

EPSC Amplitude (pA)	27.8	2.00	29/6	21.5	1.98	26/6	0.02
EPSC Frequency (Hz)	0.6	0.11	29/6	0.3	0.07	26/6	0.07

Table S2. Statistical values for Let-7c overexpression experiments using the CRM27 cell line. AP = Action Potential, Syn = Synapsin, EPSC = excitatory post-synaptic current. N: numbers of neurons/ batches of cultures

	Control			Let-7c			
	Mean	SEM	N	Mean	SEM	N	p
Soma Size (μm^2)	182	66.5	20/2	174	20.3	23/2	0.75
Number of 1° Dendrites	5.6	1.54	20/2	5.7	3.05	23/2	0.84
Spontaneous AP Incidence	0.51	0.07	4	0.05	0.05	4	0.001
Number of Evoked APs	5.2	1.20	9/2	4	0.72	7/2	0.77
Evoked AP Half-width (ms)	4.5	0.81	9/2	5.8	1.35	7/2	0.40
Evoked AP Firing Threshold (-mV)	41	1.15	9/2	40	2.21	7/2	0.56
Evoked AP Amplitude (pA)	70.6	5.32	9/2	60.1	7.43	7/2	0.26
Syn$^+$ puncta per 10μm^2 dendrite	1.59	0.13	67/5	1.59	0.15	71/2	0.98
Syn$^+$ Puncta Size (μm^2)	0.49	0.018	66/5	0.48	0.020	69/5	0.63
Syn$^+$ Puncta Intensity (a.u.)	84.3	2.85	66/5	80.0	3.17	69/5	0.33

Spontaneous EPSC Incidence	0.85	0.08	4	0.79	0.12	4	0.69
EPSC Amplitude (pA)	27.1	3.37	20/4	20.3	2.11	16/4	0.11
EPSC Frequency (Hz)	0.2	0.06	20/4	0.3	0.07	16/4	0.79

Table S3. Statistical values for Let-7c overexpression experiments using the H1 cell line. AP = Action Potential, Syn = Synapsin, EPSC = excitatory post-synaptic current. N: numbers of neurons/ batches of cultures

	Control			Let-7c			
	Mean	SEM	N	Mean	SEM	N	p
Soma Size (μm^2)	168	10.1	93/3	165	10.8	92/3	0.75
Number of 1° Dendrites	5.81	0.18	108/3	5.16	0.18	102/3	0.011
Spontaneous AP Incidence	0.63	0.06	3	0.12	0.06	3	0.006
Evoked AP Incidence (%)	0.94	0.03	3	0.68	0.05	3	0.0087
Evoked AP Rheobase (pA/pF)	0.77	0.07	36/3	1.19	0.10	33/3	0.0014
Syn$^+$ Puncta Size (μm^2)	0.47	0.014	92/3	0.47	0.020	92/3	0.90
Syn$^+$ Puncta Intensity (a.u.)	84.8	0.99	92/3	85.0	1.07	92/3	0.88
Correlated Syn$^+$ Puncta/Field	66.2	5.55	93/3	45.8	4.15	93/3	0.003
Spontaneous EPSC Incidence	0.78	0.03	3	0.76	0.03	3	0.65
EPSC Amplitude (pA)	21.1	1.11	25/3	17.9	0.96	23/3	0.038
EPSC Frequency (Hz)	1.30	0.15	25/3	0.56	0.16	23/3	0.037

Supplemental Figure

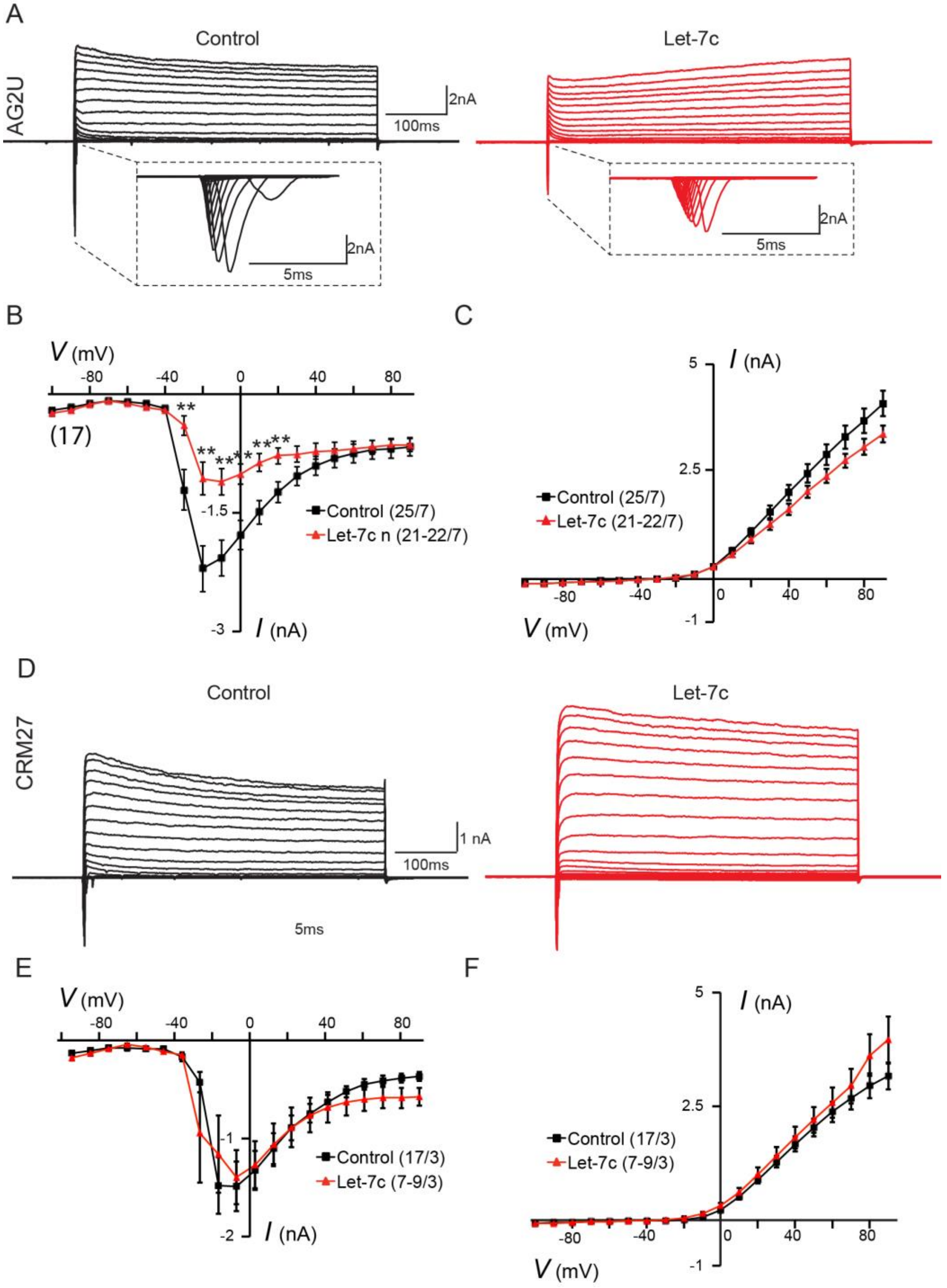

Supplemental Figure S1. Effect of Hsa-Let-7c on whole-cell sodium and potassium currents in iN cells. A-C. iN cells from the AG2U cell line that overexpress Let-7c show reduced sodium currents, but nochange in delayed rectifying potassium currents. **D-F.** iN cells from the CRM27 line that overexpress Let-7c show no change in either sodium or potassium currents. Data are presented as Mean +/-SEM and n numbers are indicated as cell numbers/culture batch numbers. Student t tests were used.
